# Balsam of Peru in Phthisis

**Published:** 1890-03-29

**Authors:** 


### The Practitioner's Mirror and Retrospect.

*[The Editor will be glad to receive offers of co-operation and contributions from members of the profession. There is no doubt that much valuable material full of interest and instruction is at present lost to science. This is largely so in the case of* (1) *the younger and less-known members of the hospital staff* (2) *those connected with cottage hospitals, and* (3) *the great body of medical practitioners not attached to any institution. The co-operation of all is cordially invited to enable the Editor to make* The Hospital *of the utmost possible use and value to the profession. Specialists willing to review books on their special subjects are invited to communicate this fact at once. All letters should be addressed to* The Editor, The Lodge, Porchester Square, London, W.]

### MEDICINE.

### BALSAM OF PERU IN PHTHISIS.

The oleoresins of several trees belonging to the totally different orders of Leguminosæ and Styraceæ, have long been used as parasiticides, as applications to unhealthy and suppurating wounds or mucous membranes (as bactericides?), and as stimulants to the respiratory tracts. They agree in containing styrone or cinnamic ether among other allied bodies, and to it they owe their properties. Within the last few years, however, Dr. J. Pauly, of Nervi, near Genoa, has turned the old-fashioned and semi-obsolete drug, Balsam of Peru, to new uses, and Dr. Landerer, of Leipzig, has followed in the same line of experiment. They began with the application of Balsam of Peru pure, or dissolved in ether, mixed as a plaster or as an emulsion with yolk of eggs, as applications to tuberculous ulcers, glands, and joints. The results were so satisfactory as to encourage them to attempt its interna administration, as an intravenous injection, with a view to avoiding the decomposition it would undergo in the digestive canal. Since an etherial solution would be too irritative, Landerer tried an emulsion in mucilage of gum arabic on frogs, but found that the particles obstructed the capillaries of the lung. He then substituted the yolk of an egg as a medium, which happily proved free from this objection. Experimenting on rabbits previously rendered tuberculous he was gratified by finding on post-mortem examination that the bacilli were greatly reduced in numbers, that tubercular foci were more or less encapsuled, and in many places had begun to calcify, a process of self-healing never otherwise observed in rabbits. Since 1885 Landerer has practically employed intravenous injections in a number of cases and with the most encouraging results. The progress of advanced tubercular phthisis, with extensive cavities, and high fever, &c., has been appreciably retarded, the patients having been enabled to resume for some time their usual occupations. A young lady with intestinal tuberculosis, passing eight to twelve bloody stools daily, with œdema of the lower extremities, fever, &c., has been so far benefited that the stools have been reduced to two daily, free from blood, while the œdema has entirely disappeared, the temperature become normal, and she has gained in weight. Two patients with suppurating fungoid disease of joints, each of whom had previously had one leg amputated for the same disease, have been completely cured for five years, and have gained 20 and 30 lbs. respectively in weight, one—a woman—having since given birth to a healthy child. Dr. Landerer injects 0·5 to 1·0 gram of the balsam of Peru twice daily, in enough yolk of egg to make a conveniently fluid emulsion.

